# A Diagnosis of Idiopathic Hypereosinophilic Syndrome With Skin Involvement in a Patient Presenting With Lymphadenopathy and Rash

**DOI:** 10.7759/cureus.23580

**Published:** 2022-03-28

**Authors:** Nishanth Sekar, Maheswaran Umakanth

**Affiliations:** 1 Internal Medicine, Faculty of Health Care Sciences, The Eastern University, Chenkalady, LKA; 2 Clinical Medicine, Teaching Hospital Batticaloa, Batticaloa, LKA

**Keywords:** hypereosinophilia syndrome, heart failure, imatinib mesylate, systemic steroids, cervical lymphadenopathy

## Abstract

Idiopathic hypereosinophilic syndrome is a rare entity where the diagnosis is made after all the probable causes of hypereosinophilia are excluded. The characteristic organ involvement includes the heart, nervous system, lung, and gastrointestinal tract. The mainstay of treatment is corticosteroids. Patients who are unresponsive to the steroids require immunomodulatory therapy that includes imatinib, mepolizumab, and in some resistant cases alemtuzumab. We describe a case of idiopathic hypereosinophilic syndrome with skin involvement without other organ infiltration in a previously unevaluated South Asian male who responded well to the initiation of steroid therapy.

## Introduction

Hypereosinophilic syndrome (HES) is one of the spectra of disorders where eosinophilic infiltration causes organ damage. HES is categorized into three main groups according to the etiology. They are primary, secondary, and idiopathic HES [1]. When the etiological workup excludes any causative agent, it falls under the idiopathic HES group. Though the major organs involved are the gastrointestinal tract, heart, lung, skin, and nervous system, the patient sometimes presents with systemic symptoms like fever or weight loss without major organ involvement where again the responsiveness to steroids is the hallmark of diagnostic confirmation [2].

Idiopathic HES is a diagnosis of exclusion. The criteria for HES include a sustained absolute eosinophil count greater than 1500/µl which persists longer than 6 months, absence of another identifiable etiology for eosinophilia, and there are signs and symptoms of organ involvement [2]. Skin lesions occur in more than 50% of the cases and can be the initial presentation [3]. Eczema, erythroderma, urticaria, angioedema along with lymphadenopathy are the common manifestations [3]. The steroid is the initial mode of therapy.

## Case presentation

**Figure 1:**
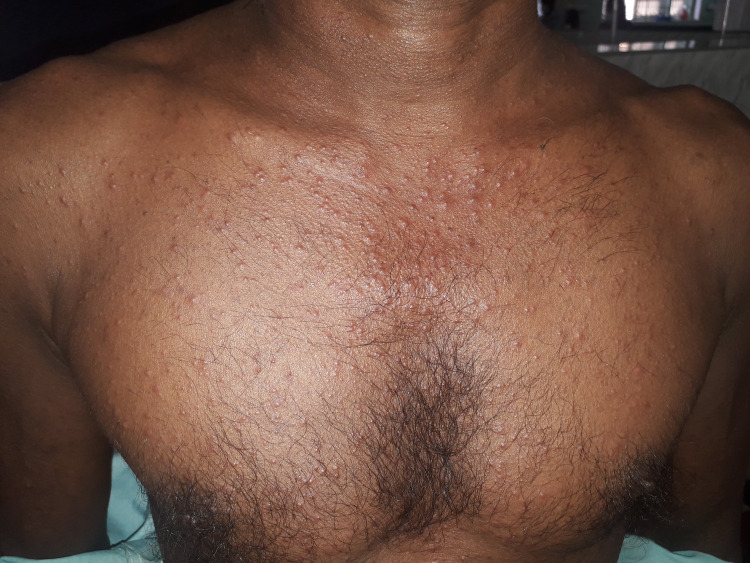
The rash on the chest

52-year-old Sri Lankan gentleman who was previously unevaluated presented with multiple erupted lumps on the neck, axilla, and groins bilaterally for 3 weeks duration with intense itching rash mainly involving the trunk for 8 months duration (Figure 1). The bumps started from the neck and progressed to the axilla and later on to the groin region bilaterally. He was afebrile throughout but experienced a significant loss of weight and loss of appetite associated with malaise and fatigue.

**Figure 2:**
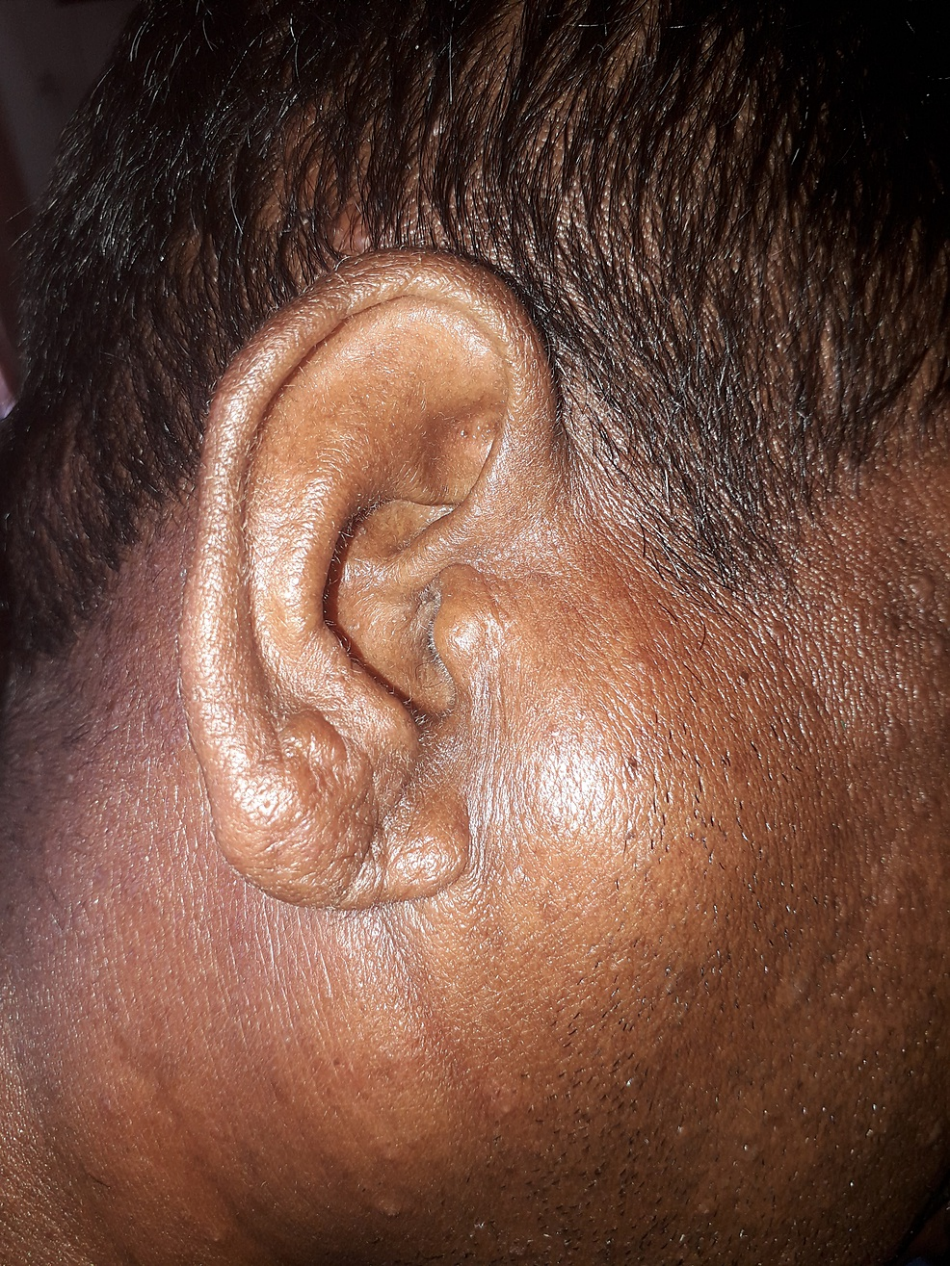
Preauricular lymph node enlargement

On the general examination, he had generalized firm non-tender cervical including the preauricular (Figure 2), submandibular, axillary, and inguinal lymphadenopathy with diffuse extensive papules on the trunk relatively sparing the limbs. A nodularity on the ear lobe was also noted (Figure 2). The abdominal examination didn’t elicit any hepatosplenomegaly. The cardiovascular, respiratory, and nervous system examinations were unremarkable.

The investigations are shown in Table 1.

**Table 1:** The investigations performed. LDH: lactate dehydrogenase; ALT: alanine transaminase; AST: aspartate transaminase; ALP: alkaline phosphatase; APTT: activated partial thromboplastin time; US abdomen: ultrasound scan of the abdomen; ESR: erythrocyte sedimentation rate; CRP: C reactive protein; INR: international normalized ratio; Serum Na+: serum sodium; Serum K+: serum potassium serum; Ca2+: serum calcium; UFR: urine full report; hpf: high-power field

Test	Reference values	Results on admission	Results after 1 week of admission
Full blood count			
White cell counts (10^3^/µl)	4-11	34.3	12.1
Neutrophils 10^3^/µl	2-7	5.1	6
Lymphocytes 10^3^/µl	1-5	2.1	3.1
Eosinophils 10^3^/µl	<0.5	22.3	1
Monocytes 10^3^/µl	0.2-0.8	4.9	2
Platelets 10^3^/µl	150-400	241	222
Hemoglobin g/dl	11-15	14.3	14
CRP mg/l	<5	35	11
ESR mm/h	<22	10	
LDH U/l	<234	250	
Serum Na^+ ^mmol/l	135-145	142	141
Serum K^+ ^mmol/l	3.5-5.1	4	3.9
Serum Ca^2+ ^mmol/l	2.1-2.6	2.4	
Serum creatinine µmol/l	100-115	104	102
Blood Urea mmol/l	3-7	5.6	5.4
ALT U/l	10-50	28	26
AST U/l	10-40	31	30
ALP U/l	25-150	63	60
Gamma-glutamyl transferase U/l	10-65	35	32
Total protein g/l	65-83	65	68
Serum Albumin g/l	35-50	40	39
Serum Globulin g/l	20-40	25	29
Total Bilirubin µmol/l	5-17	11	10.7
INR	<1.1	1	
APTT	30-40 seconds	23	
UFR: Pus cells	Nil	Occasional/hpf	
UFR: Red cells	Nil	1-2/hpf	
UFR: Albumin	Nil	Nil	
Blood culture		No growth	
Urine culture		No growth	
Blood picture		Hypereosinophilia seen. No abnormal cells	
Chest X-ray		Normal	
ECG		Sinus rhythm	
2D echocardiography		Normal valvular status and biventricular function	
US abdomen		No hepatosplenomegaly or intraabdominal lymphadenopathy	

The contract-enhanced CT (CECT) abdomen, chest, and pelvis were normal. The upper GI and lower GI endoscopy didn’ reveal any abnormalities. Tumor markers like carcinoembryonic antigen (CA 19-9), prostate specific antigen, and alpha-feto protein were done which were all normal. Antinuclear antibody (ANA), rheumatoid factor, antineutrophil cytoplasmic antibody (C-ANCA) and P-ANCA, anti-Jo antibody, and angiotensin converting enzyme level (ACE-2) level were all normal. 9 am cortisol was 15 µg/dL (10-20 g/dL). The retroviral screening was negative. Stool ova and parasite test was also negative.

**Figure 3:**
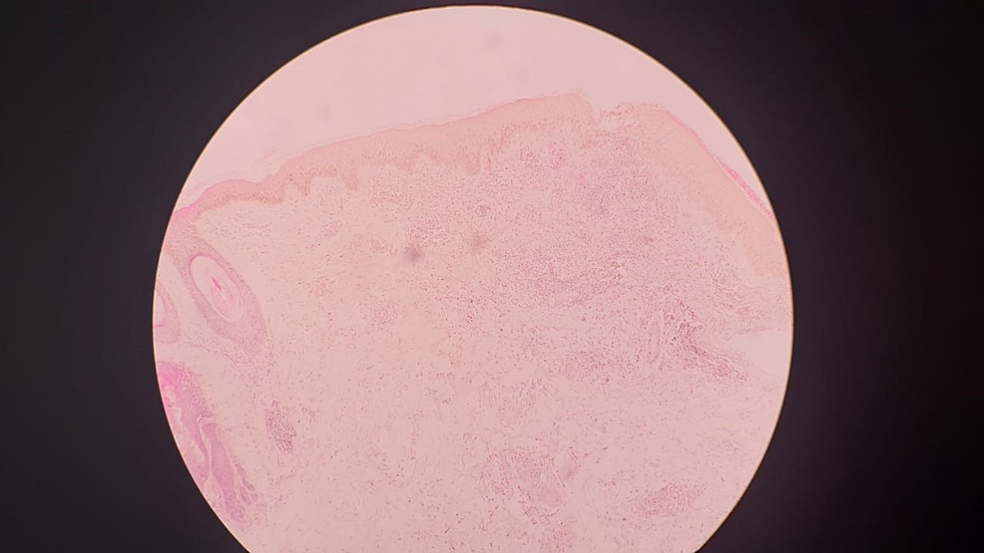
The skin biopsy from the right anterior chest wall stained with hematoxylin and eosin (×40) showing probable hypereosinophilic syndrome

**Figure 4:**
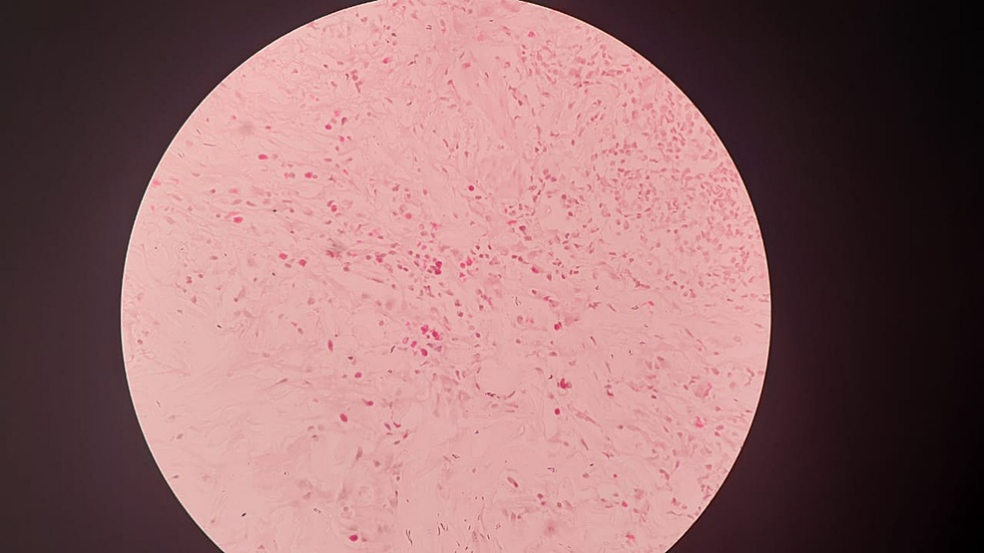
Skin punch biopsy from the left anterior chest wall stained with hematoxylin and eosin (×400) showing possible hypereosinophilic syndrome

And the skin biopsy taken from the papule on right the back of the chest (Figure 3) and left anterior chest wall (Figure 4) showed superficial dermis with heavy lymphocytic infiltrate with scattered eosinophils suggestive of HES without features of mycosis fungoides or leprosy. Mycosis fungoides were excluded due to the absence of cancer cells with twisted contour, Pautrier’s microabscesses and other histological evidence. The lymph node biopsy taken from the right upper cervical lymph node was negative for granuloma or lymphoproliferative disease. The bone marrow biopsy showed no abnormalities except high eosinophils.

**Figure 5:**
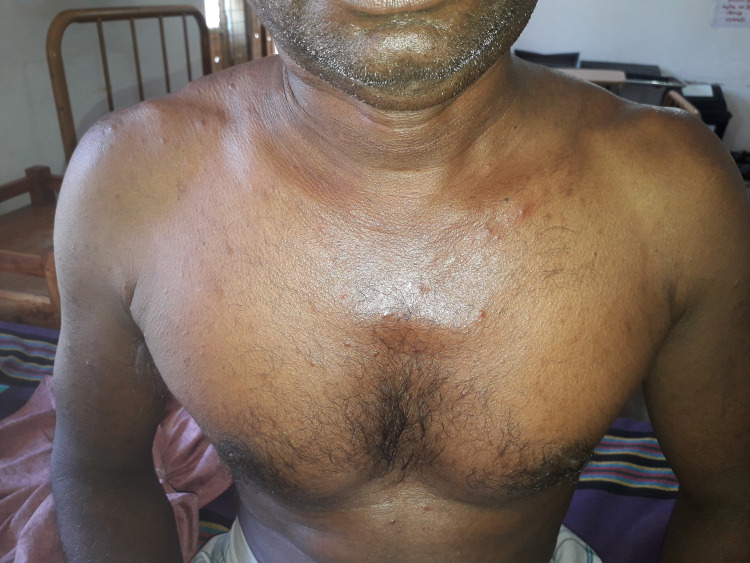
The appearance of the chest after the recrudescence of the skin rash

A multidisciplinary meeting was engineered with the inclusion of consultant hematologist, consultant dermatologist, consultant physician, and consultant histopathologist. With the multidisciplinary consensus, oral prednisolone 1 mg/kg was started and was planned to gradually taper off according to the response. After the 2 weeks completion, the patient showed a phenomenal response to corticosteroids and the lymphadenopathy and rash subsided gradually (Figure 5).

## Discussion

In our case, the symptoms of the patient have been lasting for more than 6 months with an absolute eosinophil count of 22300/µl and he had skin involvement without other major organ infiltrations. Therefore, the possible etiologies of secondary eosinophilia were excluded [4]. Several mechanisms are proposed as the pathogenic basis for the HES that includes the overproduction of the eosinophilopoietic cytokines mainly IL-3 (interleukins), IL-5, and granulocyte-macrophage colony-stimulating factor (GM- CSF) and their increased activity levels and a defect in the normal suppressive pathway of those eosinophilopoietic systems [3]. We ruled out the malignancies, connective tissue diseases, skin diseases before the diagnosis. The causes of pulmonary eosinophilia such as Loffler’s syndrome, bronchopulmonary aspergillosis, and Churg-Strauss syndrome were then excluded. We also ruled out inflammatory bowel disease, parasitic infection, and sarcoidosis which are the other known causes of hypereosinophilia [5]. FIP1L1-PDGFRA fusion which is a well-known molecular cause was not assessed due to the limitations. Management varies depending on the FIP1L1-PDGFRA mutation [6]. The presence of the mutation incurs a worse prognosis. In all patients without the aforementioned mutation, glucocorticoids are the first-line therapy.

Only one-third of cases are resistant to steroid therapy. Failing that, interferon α and hydroxyurea are tried as the second-line agents. When the response to first- and second-line agents are minimal, we opt to start high-dose imatinib (400 mg/d). Patients with FIP1L1/PDGFRA mutation need aggressive treatment. For them, the drug of choice is imatinib which is shown to have more or less 100% responsiveness [6]. Refractory cases are treated with chemotherapeutic agents like etoposide, chlorambucil, and vincristine or hematopoietic stem cell transplantation.

Though our patient didn’t develop any major organ infiltration, there are certain surgical indications for the patients with idiopathic hypereosinophilic syndrome (IHS). Endocardiectomy may be required in the endomyocardial fibrosis and thrombectomy in those patients with thrombosis. The candidates for splenectomy are those with splenic infarction and hypersplenism [2]. The success of the therapy can be assessed by the clinical improvement along with the hematological improvement. There would be a drastic amelioration in the blood eosinophilia and therefore paves a way to assess the response. Mostly, the HIS cases are described all around the world with mainly cardiac and gastrointestinal involvement [1]. In addition, there are some case reports of patients with HES presenting with embolic stroke [7]. In our case, the patient only presented with lymphadenopathy with skin manifestations therefore the absence of gastrointestinal, lung, and cardiac involvement doesn’t necessarily discourage the diagnosis of IHS.

## Conclusions

It is always important to be borne in mind that idiopathic HES is a diagnosis of exclusion when encountered with a case of hypereosinophilia. It can present as lymphadenopathy with mere skin involvement without major organ infiltration. Corticosteroid therapy is the initial management and shows a phenomenal treatment response in most of the cases. 

## References

[1] Abo Shdid R, Azrieh B, Alebbi S, Mansour S, Naeem M (2021). Idiopathic hypereosinophilic syndrome with multiple organ involvement. Case Rep Oncol.

[2] (2021). Hypereosinophilic Syndrome. https://emedicine.medscape.com/article/202030-overview.

[3] (2021). Hypereosinophilic Syndrome (Idiopathic hypereosinophilic syndrome). https://www.dermatologyadvisor.com/home/decision-support-in-medicine/dermatology/hypereosinophilic-syndrome-idiopathic-hypereosinophilic-syndrome/.

[4] (2022). Hypereosinophilic Syndromes: Clinical Manifestations, Pathophysiology, and Diagnosis. https://www.uptodate.com/contents/hypereosinophilic-syndromes-clinical-manifestations-pathophysiology-and-diagnosis.

[5] Sumana D (2018). Eosinophilia. Oxford medicine.

[6] Roufosse F (2009). Hypereosinophilic syndrome variants: diagnostic and therapeutic considerations. Haematologica.

[7] Silva MS, Ramalho C, Ferreira F, Maia I, Joosten A (2021). Idiopathic hypereosinophilic syndrome presenting with embolic stroke. Cureus.

